# Promoting Effect of Ball Milling on the Functionalization and Catalytic Performance of Carbon Nanotubes in Glycerol Etherification

**DOI:** 10.3390/molecules29071623

**Published:** 2024-04-04

**Authors:** Karolina Ptaszyńska, Anna Malaika, Katarzyna Morawa Eblagon, José Luís Figueiredo, Mieczysław Kozłowski

**Affiliations:** 1 Faculty of Chemistry, Adam Mickiewicz University in Poznań, Uniwersytetu Poznańskiego 8, 61-614 Poznań, Poland; amalaika@amu.edu.pl; 2 LSRE-LCM—Laboratory of Separation and Reaction Engineering—Laboratory of Catalysis and Materials, Faculty of Engineering, University of Porto, Rua Dr. Roberto Frias, 4200-465 Porto, Portugal; keblagon@fe.up.pt (K.M.E.); jlfig@fe.up.pt (J.L.F.); 3 ALiCE—Associate Laboratory in Chemical Engineering, Faculty of Engineering, University of Porto, Rua Dr. Roberto Frias, 4200-465 Porto, Portugal

**Keywords:** carbon nanotubes, surface functionalization, ball milling, glycerol etherification, fuel additives

## Abstract

A facile and eco-friendly approach using in situ-generated 4-benzenediazonium sulfonate (BDS) was applied to prepare highly functionalized carbon nanotubes (CNTs). The effectiveness of this functionalization was additionally enhanced by a green and short-time ball milling process applied beforehand. The obtained BDS-modified CNTs presented significant activity in glycerol etherification, producing tert-butyl glycerol ethers, which are considered promising fuel additives. Excellent results of ~56% glycerol conversion and ~10% yield of higher-substituted tert-butyl glycerol ethers were obtained within just 1 h of reaction at 120 °C using a low catalyst loading of only 2.5 wt.%. Furthermore, the sulfonated CNTs were reusable over several reaction cycles, with only a minor decrease in activity. Additionally, the sample activity could be restored by a simple regeneration approach. Finally, a clear correlation was found between the content of -SO_3_H groups on the surface of CNTs and the catalytic performances of these materials in glycerol etherification. Improved interaction between functionalized ball-milled CNTs and the reactants was also suggested to positively affect the activity of these catalysts in the tested process.

## 1. Introduction

Due to their unusual properties, such as large surface area, low toxicity, high thermal and electrical conductivity, excellent mechanical strength, and the possibility of surface modifications, carbon nanotubes (CNTs) have gained significant attention in various branches of industry, being used as promising materials for flexible electronics, biomedical applications, adsorption processes, energy storage devices, water purification systems, or catalysis, among others [1,2,3,4]. Additionally, due to the continuous development of carbon nanotube synthesis methods and the possibility of their efficient production, the prices of these materials are constantly decreasing, making them more and more competitive with other materials used in industry [5].

Typically, CNTs in their raw form are scarcely used, and their functionalization is required to boost their applications. For example, Chen et al. [6] found raw multi-walled CNTs ineffective in adsorbing Hg(II) from water. At the same time, the sample performance was significantly enhanced by functionalization with the -COOH or -OH groups. Pantarotto et al. [7] showed that ammonium-functionalized CNTs form supramolecular complexes with plasmid DNA that can penetrate the cell membranes, working as advanced delivery systems for therapeutics. In turn, Eblagon et al. [8] applied CNTs as supports for Au and obtained an efficient bifunctional catalyst for the direct conversion of cellobiose to gluconic acid, which outperformed Au supported on carbon xerogel or on ordered mesoporous carbon. 

It should be taken into account that the functionalization of CNTs is challenging due to their rigid and inert structure [9,10]. For example, Wei et al. [11] introduced only 1.2% sulfur into the structure of multi-walled carbon nanotubes by sulfonation. Yu et al. [12] performed a two-step functionalization of CNTs by treating the sample with a mixture of 1:1 concentrated HNO_3_ and HCl, followed by a reaction with H_2_SO_4_ at elevated temperatures up to 300 °C, and obtained a final acid density of only 0.67 mmol/g. In turn, Nowicki et al. [9] reported that the susceptibility of carbon nanotubes to oxidation with nitric acid was three times lower than that of activated carbon.

Cutting or crushing CNTs is an interesting method of changing their structural and morphological properties and, consequently, their chemical features. This approach breaks and opens the closed ends of CNTs, making the interior accessible for various atoms and molecules [13,14]. Furthermore, the mechanical pretreatment of CNTs can increase the number of exposed active graphene edges, thus activating the sample toward functionalization [15]. 

Several approaches for mechanical modification of CNT structure and morphology have been described in the literature [13,16]. Among them, the most useful option seems to be ball milling (BM), which proved to be a simple, reagent-free, effective, versatile, controllable, and reproducible method. Furthermore, BM can be applied under wet and dry conditions to a wide range of materials [16,17,18,19]. For example, Pierard et al. [18] showed that vibratory ball milling was effective in decreasing the single-walled carbon nanotube length, and adjusting the processing time enabled to vary the structural disorders and defects in the CNTs (showed by different intensity ratios between the D- and G-bands in Raman spectra). On the other hand, Soares et al. [19] found that the particle sizes and S_BET_ of CNTs could be controlled by changing the ball-milling vibration frequency. The obtained ball-milled samples showed an increased activity in the ozonation of oxalic acid due to changes in their textural properties. Gharegozloo et al. [20] found that a BM strategy can prepare effective Ni-CNT composite catalysts. In turn, Soares et al. [21] proposed a simple two-step mechanochemical method for functionalizing CNTs with heteroatoms, involving ball-milling of CNTs in the presence of adequate precursor and subsequent thermal treatment under an inert atmosphere. The ball milling allowed the creation of active sites for functionalization and homogenization of the mixture. In the following thermal step, the precursor was thermally decomposed, and the heteroatoms were attached to the previously created defects on the surface of CNTs. A ball milling strategy was also used to obtain CNT-polymer composites, enhancing the adhesion between CNTs and a polymer matrix and yielding samples with enhanced thermal, mechanical, or chemical properties [22].

Sulfonic groups (i.e., SO_3_H)-functionalized carbons (so-called solid acids) are of special interest for acid-catalyzed reactions [23,24]. These catalysts are typically obtained via the sulfonation of samples with concentrated sulfuric acid, chlorosulfonic acid, or their mixtures [24,25,26]. However, to achieve a satisfactory SO_3_H-functionalization degree of CNTs, harsh reaction conditions and/or aggressive reagents are generally required [27,28]. In the current work, a benign approach applying diazonium salt generated in situ was employed to produce green solid acids by anchoring Ph-SO_3_H groups onto the surface of CNTs. Additionally, the effect of ball milling on the susceptibility of CNTs to sulfonation via arylation was studied for the first time. The obtained SO_3_H-functionalized CNTs were applied as catalysts in glycerol etherification with TBA (tert-butyl alcohol) to produce tert-butyl glycerol ethers (TBGEs) via a green synthesis route. Active catalysts for this reaction are of great industrial interest because TBGEs are valuable oxygenated fuel additives that can limit particulate matter and NO_x_ emissions [29]. The relationship between the chemical nature of CNTs-SO_3_H and their catalytic performances toward the formation of TBGEs was established in this work. Recyclability tests were also carried out, and a simple method was proposed to recover the catalytic activity of the spent catalysts.

We believe the findings presented in the paper shed new light on the possible applications of CNTs as solid acid catalysts and lay the groundwork for developing other effective catalysts for the synthesis of sustainable fuel additives.

## 2. Results and Their Discussion

### 2.1. Characterization of the Samples

**Figure 1:**
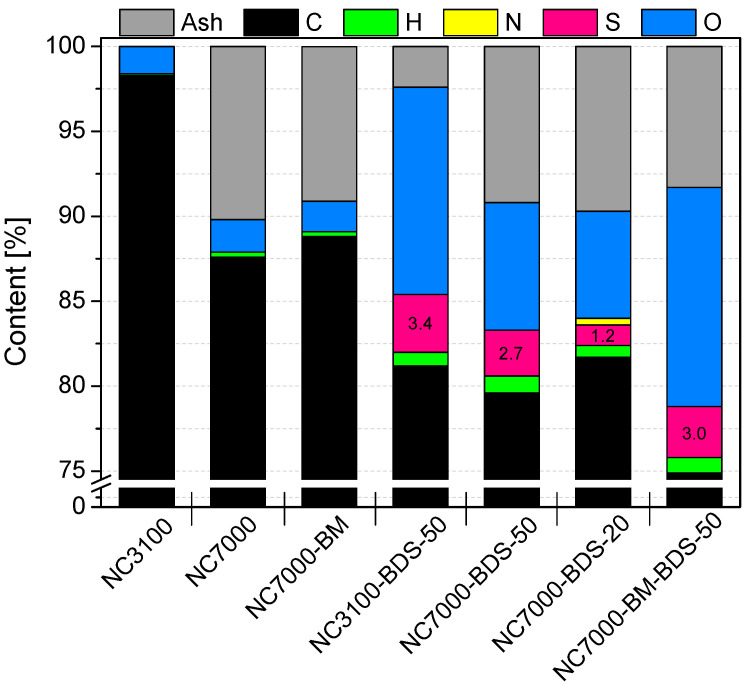
The results of elemental and ash analyses obtained for the raw, ball-milled, and modified carbon nanotubes.

The results of elemental analysis and the amount of ash obtained for the tested nanotubes are presented in Figure 1. As observed, the raw carbon nanotubes, i.e., NC3100 and NC7000, differed quite significantly in their compositions. Elemental carbon was dominant for both materials; however, the NC7000 sample contained ~10% less C than NC3100. Instead, NC7000 indicated about 10% of ash, which was probably related to the presence of the metal catalysts used during the production process (i.e., catalytic chemical vapor deposition (CCVD)) that were not removed in the purification step [30]. On the contrary, NC3100 was free from inorganic residues (0% ash), suggesting thorough material purification after the CCVD. In both raw CNT samples, the oxygen and hydrogen contents were negligible.

As observed in Figure 1, the modifications of CNTs with diazonium salt introduced significant amounts of sulfur and oxygen to the structure of the raw materials. The content of S in the modified samples was between 1.2 and 3.4%, which is quite impressive considering the efficiency of sulfonation of CNTs reported in the literature. For example, Koskin et al. [31] used concentrated H_2_SO_4_ to treat CNTs at 200 °C and obtained only 0.15% sulfur content in the sample. The modification of CNTs with fuming sulfuric acid (oleum) was more effective as it introduced 0.89% of S to the material; however, this process also required a high temperature (175 °C). Furthermore, our previous studies showed that only about 0.5–0.6% of S could be introduced into CNTs when using concentrated H_2_SO_4_ at 180 °C [32]. Given the above, the S content results obtained here may suggest that modification with 4-benzenediazonium sulfonate is a particularly suitable option for the efficient functionalization of CNTs. Moreover, the method has additional advantages due to mild reaction conditions applied during the treatment (see Experimental). The conclusions drawn here align with those of other authors [10].

Interestingly, NC3100 (i.e., high-purity CNTs) modified with BDS at 50 °C contained more sulfur than its technical grade counterpart, i.e., NC7000 (3.4 vs. 2.7% of S, respectively). Apparently, the metallic impurities present in NC7000 hampered the functionalization. It was also observed that the lower temperature used for the reaction with BDS, i.e., 20 °C, was not favorable for modification of CNTs (compare the S content in NC7000-BDS-20 and NC7000-BDS-50 in Figure 1), even though it was very efficient in functionalization of activated carbon, as shown in our previous work [33]. Interestingly, in the case of NC7000-BDS-20, a certain amount of nitrogen was also detected, indicating the presence of a small number of azo bonds formed in coupling reactions between diazonium cations and the surface of CNTs [24,33].

As stated above, CNTs are quite resistant to typical functionalizations. Thus, simple, reagents-free mechanical pretreatment of CNTs by BM was suggested to enhance their activity towards chemical modification. Indeed, comparing the results of S contents for NC7000-BDS-50 and NC7000-BM-BDS-50 in Figure 1, 11% higher S content was obtained for the latter sample, which clearly demonstrates that BM improved the susceptibility of NC7000 to functionalization, despite the short ball milling time applied (see Experimental). Importantly, the modifications of CNTs with diazonium sulfonate resulted not only in an increase in the S contents but also in the oxygen amounts (up to ~13%). This observation suggests that sulfur was introduced onto the sample surface as -SO_3_H groups.

Table 1 presents the acidic properties of the tested CNTs. As can be observed, the raw and ball-milled samples showed only negligible total acidities (A_tot_), probably related to a small number of acidic oxygen groups on the carbon surfaces (see also the oxygen content in Figure 1). A significant increase in the number of the -SO_3_H group and A_tot_ was noted for all the modified samples, indicating that the functionalization was effective. Interestingly, in most cases, the values of A_tot_ and calculated contents of -SO_3_H groups were not closely correlated. In the case of raw CNTs modified with BDS at 50 °C, the total acidity of the functionalized sample was lower than the calculated acidity induced by the -SO_3_H groups. We also obtained similar results when modifying activated carbons or carbon fibers with BDS [24,33]. Such a phenomenon can occur when zwitterions (i.e., internal salts) are formed, resulting in the neutralization of -SO_3_H sites [34]. The other possibility is the formation of a thick and tight layer of sulfonic species with restricted accessibility [35]. The possible surface structures formed during the modification of CNTs with BDS [36,37] are shown in Figure S1 in Supplementary Materials. The opposite effect (i.e., A_tot_ > content of -SO_3_H sites) was observed for the ball-milled NC7000 reacted with BDS at 50 °C (i.e., NC7000-BM-BDS-50 sample), indicating the presence of not only sulfonic groups on the sample surface but also small amounts of other types of acidic sites, most likely oxygen functionalities [24].

**Figure 2:**
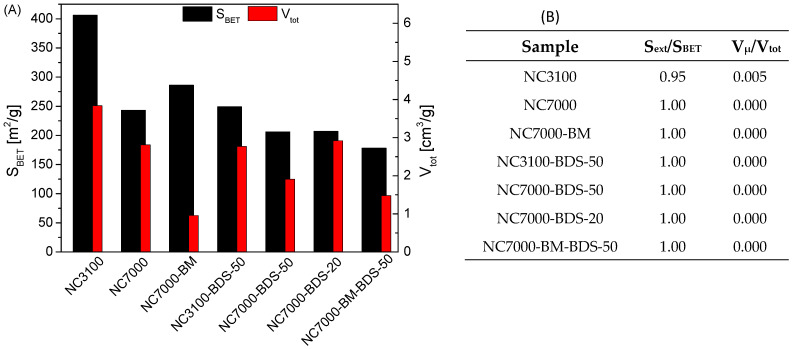
Results of the textural analysis performed for the raw and modified CNTs: (**A**) apparent surface areas of samples and total volume of pores; (**B**) contributions of external surface areas to the apparent surface areas and micropore volumes to the total volumes of pores.

Figure 2A, B depict the textural properties of the studied materials. As shown in Figure 2A, the raw carbon nanotubes, i.e., NC3100 and NC7000, differed significantly. Namely, the former presented a considerably higher S_BET_ than NC7000 (460 m^2^/g vs. 250 m^2^/g), which can be linked to the extensive post-synthesis washing of NC3100, resulting in the more accessible pores in this sample. A similar phenomenon was also observed elsewhere [38]. Interestingly, as shown by the data gathered in Figure 2B, for both raw CNTs, the S_BET_ parameters resulted mainly from the external surface areas (S_ext_), i.e., meso- and macropores, and the samples’ porosity was probably related mostly to certain spaces between nanotubes [8,39]. 

Interestingly, ball milling increased the S_BET_ of NC7000, most likely due to agglomerates’ fragmentation, shortening of the tubes, and caps opening during the mechanical treatment [19,39]. On the contrary, a drastic drop in the V_tot_ of NC7000 was observed after BM, which was probably the result of the CNTs colliding with the balls and the container walls, reducing the distance between the nanotubes in bundles [39]. A significant drop in all the textural parameters was observed in modified samples compared with the as-received CNTs, which suggested the successful introduction of the functional groups blocking the surfaces of the samples [34]. 

**Figure 3:**
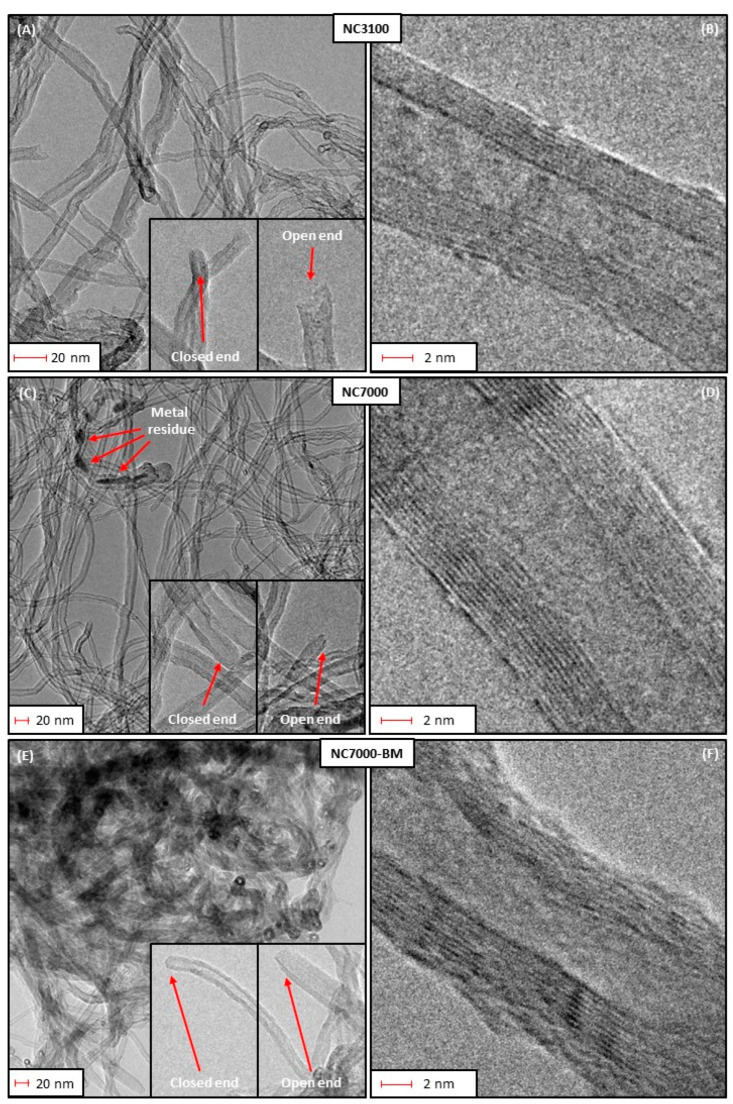
The high-resolution transmission electron microscopy (HRTEM) images of: (**A**–**D**) the as-received, (**E**,**F**) ball-milled CNTs.

Figure 3 depicts high-resolution TEM images obtained for the raw and ball-milled CNTs. As observed in Figure 3A, research-grade NC3100 consisted of randomly organized and long nanotubes that showed small diameters with varied sizes. The presence of closed caps and open ends is visible in these micrographs. The images also suggest that the NC3100 sample was free of metallic impurities or amorphous carbon. Figure 3B, presenting the NC3100 material at higher magnification, revealed that a single carbon nanotube consisted of several perfectly arranged graphene sheets parallel to each other, thus representing the structure typical for multi-walled carbon nanotubes (MWCNTs). The morphology of a technical grade NC7000 (Figure 3C) was similar to that of the research-grade NC3100. The most important difference between these two types of CNTs was the presence of impurities (the CCVD catalyst mentioned before) trapped inside the NC7000 structure, observed as dark spots in Figure 3C. This finding also aligns with the increased ash in this sample (see Figure 1). Residues of the CCVD catalyst trapped in the CNT structure were also observed in TEM images presented elsewhere [40,41]. 

Similarly to NC3100, NC7000 also indicated a multi-walled CNT-type structure; however, the average number of walls seemed higher in the latter than in the former (Figure 3D). Figure 3E shows the TEM micrographs obtained for the ball-milled sample, i.e., NC7000-BM. Significant changes in the morphology are visible before and after the ball milling process (compare Figure 3C,E). Namely, carbon nanotubes were still visible after BM; however, they formed tight bundles, probably resulting from the collisions of tubes with balls during milling. This observation also agrees well with the decreased V_tot_ obtained for the ball-milled sample (shown in Figure 2A). Moreover, only small deformations of the arrangement of graphene layers of the tubes were observed after ball-milling (shown in Figure 3F), which confirms the high mechanical stability of CNTs [42] and relatively mild BM conditions applied here (see Experimental). 

SEM images of the raw and ball-milled CNTs are presented in Figure S2 in the Supplementary Materials. As observed, the raw sample showed a rather compact structure formed by long, tightly entangled fibers clumped into large agglomerates (Figure S2A). In turn, ball milling resulted in significant fragmentation of the CNT agglomerates, leading to more exposed particle edges (Figure S2B). This was probably the reason for the enhanced S_BET_ of NC7000-BM and a higher degree of its functionalization compared with NC7000 (compare results for NC7000-BM-BDS-50 and NC7000-BDS-50 in Figure 1 and Figure 2 and Table 1).

**Figure 4:**
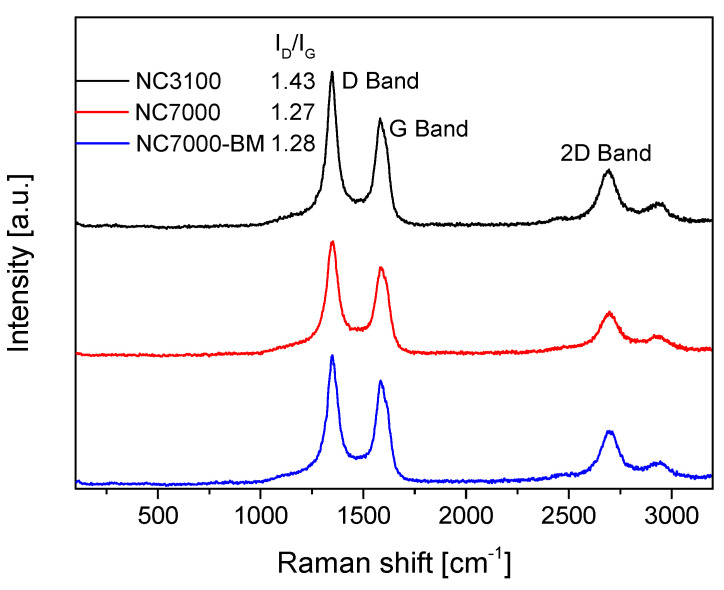
The Raman spectra obtained for the raw and ball-milled samples.

Raman spectroscopy is a useful method to investigate the effect of ball milling on the structural integrity of CNTs, as demonstrated by Pierard et al. [18]. The Raman spectra obtained for the raw and ball-milled samples tested in this study are collected in Figure 4, together with the I_D_/I_G_ ratios for the selected samples. As can be seen in this figure, all of the samples showed intense signals at 1350 cm^−1^, i.e., a D band typically assigned to disordered forms or structural defects, and at ~1580 cm^−1^, i.e., a G band corresponding to in-plane vibrations of C sp^2^ atoms and attributed to the ordered graphite structure [43,44]. Interestingly, research-grade NC3100 showed a lower degree of ordering and a higher number of defects than the NC7000 sample. Presumably, this could be related to the post-treatment purification process applied to NC3100 (to remove the CCVD catalyst), which led to the appearance of structural defects in this sample. Similar findings were reported by other authors [45,46]. As observed, the mechanical pretreatment of NC7000 did not result in significant changes in the structural ordering of the raw material, as the I_D_/I_G_ ratio obtained for NC7000-BM was only slightly higher than that observed for NC7000. This is in line with the TEM and SEM results, suggesting that ball milling resulted in the fragmentation of the agglomerates and did not affect the arrangement of the graphene layers; thus, the crystallinity of the CNTs was maintained. Li et al. [47] also did not notice an increase in I_D_/I_G_ achieved for ball-milled MWCNTs despite some structural deformations of the modified sample observed in the TEM images. In turn, Chebattina et al. [48] obtained an increased I_D_/I_G_ ratio for the mechanically treated CNTs only after an extended period of ball-milling (i.e., 20 h).

The TG analysis of raw CNTs was performed under airflow, and the DTG results are presented in Figure S3 in Supplementary Materials. As observed, the DTG patterns of both samples showed an intense peak with a minimum at ~680 °C, typically observed in multi-walled CNTs [49]. Furthermore, the DTG profile of NC7000 had a poorly separated low-intensity peak at the temperature of ~600 °C. It cannot be excluded that, in this case, the combustion of the sample was facilitated by the contaminants present in NC7000 (see also the ash amount in NC7000 in Figure 1). Kumar et al. [50] also observed that oxidation of carbon in Co_3_O_4_/CNTs nanocomposites occurred at lower temperatures than in the case of pristine CNTs. 

**Figure 5:**
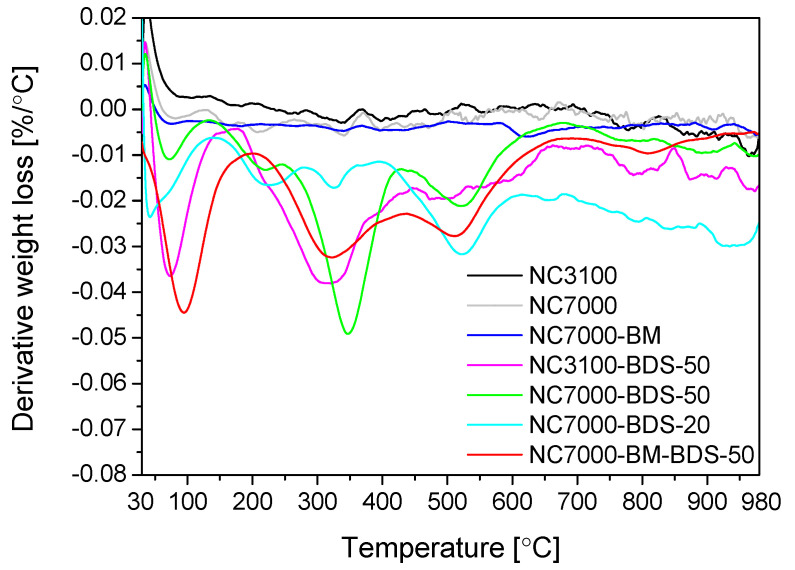
The DTG patterns of the raw and modified samples (N_2_ atmosphere).

TG analysis performed under an inert gas flow (nitrogen) can reflect the effectiveness of sample functionalization [24]. Figure 5 presents the TG results obtained for the raw and modified NC3100 and NC7000 samples (as DTG patterns). In the case of the pristine and ball-milled materials, no significant signals were observed, suggesting a very low number of surface functionalities in these samples. These results also align with data from EA (see Figure 1). As shown, the DTG profiles of the BDS-modified CNTs were significantly different from those of the parent materials, suggesting remarkable changes in the surface properties of CNTs after their functionalizations. In general, all the curves showed a peak with a minimum in the temperature range of 50–100 °C, typically attributed to moisture. Another signal at 180–260 °C was most likely related to the interlayered water evaporation [50]. However, the decomposition of -SO_3_H groups can also occur at similar temperatures [33]. In turn, the signals appearing at 300–350 °C and 500–550 °C can be associated with the gradual decomposition of the -PhSO_3_H functionalities, proceeding in two stages and related to de-sulfonation process and possibly to slight fragmentation of the benzene ring [51,52]. The peak with a minimum at 350 °C or a broad signal starting at 500 °C can also indicate the presence of oxygen species with different thermal stabilities, e.g., carboxylic anhydrides or phenolic groups [53].

**Figure 6:**
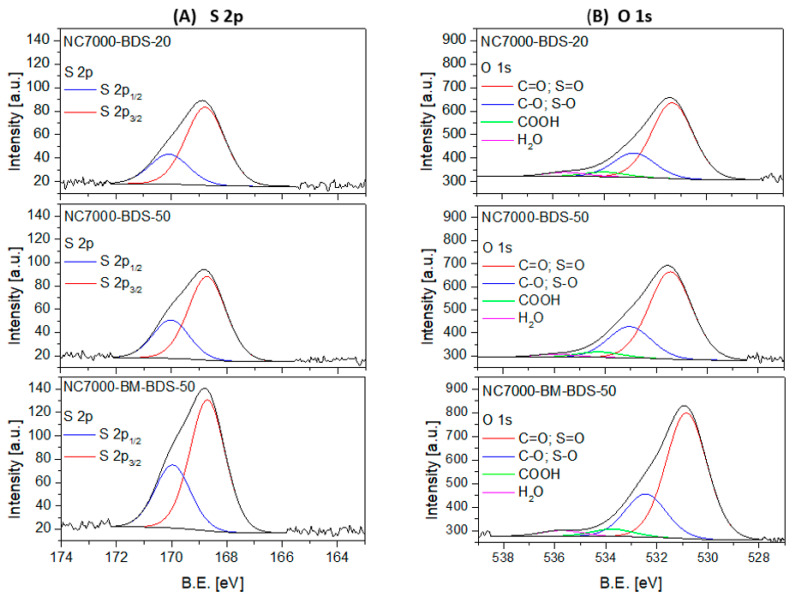
The high-resolution XPS S 2p (**A**) and O 1s (**B**) spectra of selected samples.

The XPS technique was applied to define the chemical composition of the samples’ surfaces, and the results obtained for selected modified CNTs are presented in Table 2 and Table 3 and Figure 6.

The achieved results confirmed the presence of carbon, oxygen, and sulfur in the functionalized samples, and the relative concentrations of these elements on the samples’ surfaces are presented in Table 2. All the samples contained quite a high amount of sulfur (3.9–6.8%). These quantities were higher than those obtained by EA, which suggests that S-containing moieties are more concentrated on the surface of these samples than in the bulk. Interestingly, the functionalization of CNTs with BDS was strongly affected by the temperature of the treatment. The increase in the temperature from 20 to 50 °C resulted in a 0.7% increment in the S content. Moreover, the pretreatment of CNTs via ball-milling before the functionalization allowed the incorporation of 2.2% more sulfur into the material structure. Importantly, an increase in the S content in the modified CNTs was also accompanied by an increase in the amount of oxygen, which is also in line with the EA results (Figure 1), suggesting that S was introduced in the oxidized form onto the surface of CNTs. 

The chemical and electronic states of O and S were investigated by analyzing the high-resolution XPS O 1s and S 2p spectra. 

Figure 6A presents the deconvoluted XPS S 2p spectra obtained for the modified samples. As observed, raw data was fitted with a doublet at B.E. ~168.7 eV and ~169.9 eV with an intensity ratio of 1:2 and a peak separation of 1.2 eV for all the samples, which was assigned to sulfur in -SO_3_H moieties [54]. Importantly, no signals attributed to other S species were present in these materials, showing that the applied functionalization method selectively introduces S as sulfonic groups into the CNTs.

The deconvoluted high-resolution XPS O 1s spectra shown in Figure 6B revealed the presence of four signals belonging to different oxygen species. Two intense signals at B.E. of ~531 eV and ~532.6 eV were observed in each case, which can be ascribed to C=O and C-O bonds, respectively. The signals from sulfur in S=O and S-O can also be present in this region. Additional two small peaks were present in O 1s spectra at a BE ~534.2 eV and ~535.8 eV, which are typically attributed to -COOH groups and adsorbed H_2_O, respectively [33,55,56].

The relative contributions of different sulfur or oxygen species detected in the BDS-modified CNTs are gathered in Table 3. The presented results show that the studied samples contained only oxidized forms of S in the form of -SO_3_H functionalities. This observation is also in line with our previous findings [34]. Interestingly, similar relative contributions of individual O-species were observed for each sample, i.e., a dominant contribution of C=O and S=O species (68.1–69.7%), significant of C-O and S-O (22.2–24.6%), and negligible of COOH (<1%). There were, however, some differences in the concentrations of the abovementioned species expressed in wt.%, resulting from the effectiveness of functionalizations.

### 2.2. Catalytic Results

**Figure 7:**
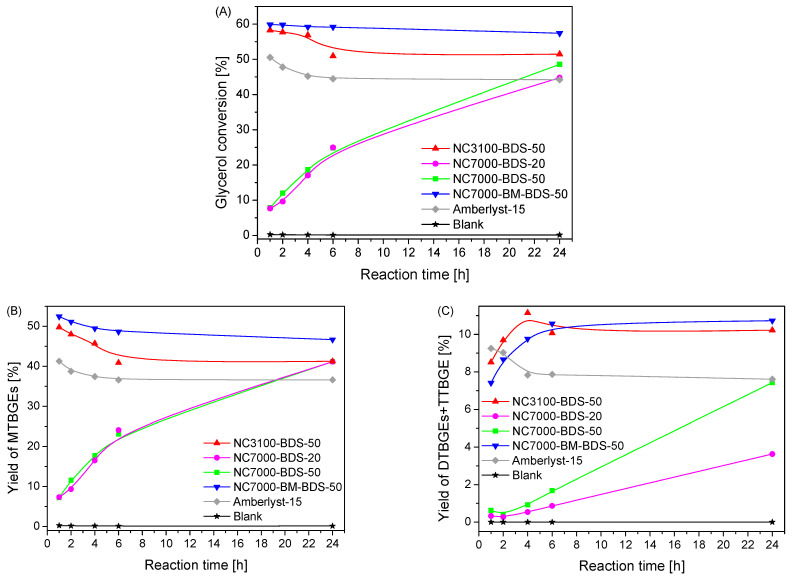
Results of the catalytic performance, i.e., glycerol conversion (**A**), yield of MTBGEs (**B**), and yield of DTBGEs+TTBGE (**C**), of the BDS-modified CNTs compared with the blank test and a reaction over Amberlyst-15 (temp. = 110 °C, G:TBA = 1:4, catalyst loading = 5 wt.%).

The catalytic performances of BDS-modified CNTs were investigated in the reaction of glycerol with tert-butyl alcohol (TBA). This process leads to mono-, di-, and tri-tert-butyl glycerol ethers (designated here MTBGEs, DTBGEs, and TTBGE, respectively); see Figure S4 in Supplementary Materials. For comparison, a blank test (reaction without a catalyst) and a reaction in the presence of a commercial catalyst, Amberlyst-15, were also performed. The obtained results are presented in Figure 7.

As presented, a reaction without a catalyst did not occur, as the final glycerol conversion (i.e., measured after 24 h) was negligible (~0.2%). The same was observed in the case of unmodified CNTs (results not shown for clarity) due to a lack of active sites for the etherification process, as confirmed by the characterization of the samples in Figure 1 and Table 1. A significant increase in glycerol conversion and the yield of TBGEs was observed with functionalized samples. Two different activity patterns were shown by the modified catalysts, as described below.

Glycerol conversions (X_G_) obtained in glycerol etherification over the produced samples are gathered in Figure 7A. As seen from this figure, the lowest glycerol conversion was displayed by the samples based on technical grade NC7000 (i.e., NC7000-BDS-20 and NC7000-BDS-50), which initially obtained rather low X_G_ (~8% within h of the process). However, a gradual increase in X_G_ was observed with time for both these catalysts, which finally presented X_G_ of 44% and 48% in the extended reaction time of 24 h. On the other hand, the remaining catalysts, i.e., BDS-modified research grade NC3100 (i.e., NC3100-BDS-50) and functionalized ball-milled NC7000 (i.e., NC7000-BM-BDS-50), were much more active in the reaction, showing a very high ~60% conversion of glycerol just within 1 h, which remained stable until the end of the testing time (due to reaching the reaction equilibrium). Only in the case of NC3100-BDS-50 was a slight decrease in X_G_ with reaction time observed, which was probably related to the subsequent de-etherification process [57]. A similar pattern of X_G_ versus time was also observed for Amberlyst-15. It should be noted that the reference catalyst showed worse catalytic performance in glycerol etherification than BDS-functionalized CNTs developed in the present work.

The excellent catalytic performances shown by NC3100-BDS-50 and NC7000-BM-BDS-50 were due to the effective functionalization, resulting in the high total acidities (A_tot_) induced mostly by -SO_3_H moieties (see results in Table 1, Table 2 and Table 3 and Figure 6). The role of A_tot_ in the activity of samples is clear when analyzing the catalytic performance of NC7000-BDS-20 and NC7000-BDS-50, which showed the lowest A_tot_ among the prepared materials (see also the discussion about the results presented in Table 1). Furthermore, it should be underlined that the catalytic performance of the modified ball-milled CNTs (i.e., NC7000-BM-BDS-50) was better than that of the functionalized raw sample (i.e., NC7000-BDS-50), which can be attributed to the higher degree of functionalization of NC7000-BM (see also Figure 1 and Table 1). It is also suggested that the mechanical pretreatment of the sample, resulting in the fragmentation of CNT particles (see SEM images in Figure 1 SM), could improve the interaction between the catalyst and the reactants in the etherification of glycerol [13].

As shown in Figure 7B,C, mono-, di-, and tri-tert-butyl glycerol ethers were detected in the reaction mixture; however, their yields (Y) varied significantly between catalysts. In each case, mono tert-butyl glycerol ethers (MTBGEs) were the dominant products. Nevertheless, significant yields of higher substituted ethers (i.e., DTBGEs + TTBGE) were also formed. The lowest yields of products (i.e., MTBGEs + DTBGEs +TTBGE) were achieved over the NC7000-BDS-20 and NC7000-BDS-50 catalysts, especially in the first hours of the process. Interestingly, similar yields of MTBGEs were obtained over time using both of these catalysts, whereas the obtained Y_DTBGEs+TTBGE_ varied between the samples (i.e., NC7000-BDS-50 gave higher Y_DTBGEs+TTBGE_). The highest production of ethers was achieved over the NC3100-BDS-50 and NC7000-BM-BDS-50 catalysts, probably due to the significant surface acidities of these catalysts (see A_tot_ in Table 1). Concerning the Y_MTBGEs_, the maximum value of over 50% was observed after 1 h. In the extended reaction time, Y_MTBGEs_ slightly decreased, which was accompanied by a simultaneous increase in the yield of higher-substituted ethers (Y_DTBGEs+TTBGE_ up to ~11%). Thus, it can be concluded that the DTBGEs and TTBGE were obtained in consecutive reactions from MTBGEs intermediate products, which is consistent with the reaction mechanism proposed by other authors [29,58]. It is worth noting that after 6 h of the process, the yields of the products stabilized due to reaching the reaction equilibrium [57]. 

**Figure 8:**
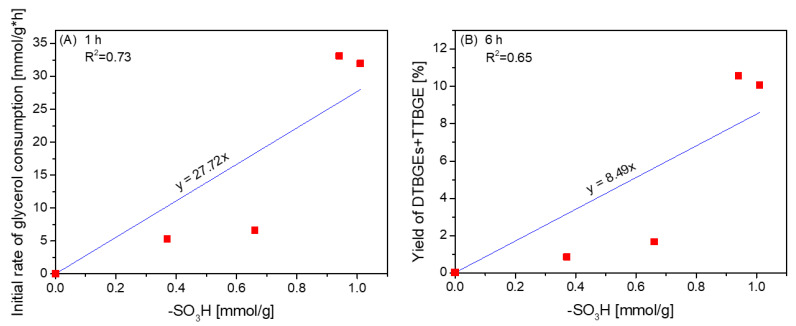
The dependence between the initial glycerol consumption rates (**A**) or yields of DTBGEs+TTBGE (**B**) and the content of -SO_3_H groups on the CNT surface.

As shown above, the catalytic performances of CNTs tested in glycerol etherification were due to the chemical properties of their surfaces, affecting X_G_ and yields of ethers. The relationship between the initial rates of glycerol consumption or yields of DTBGEs+TTBGE and the content of -SO_3_H groups present in CNTs is depicted in Figure 8. Due to the probability of zwitterions’ formation and other effects limiting the availability of sulfonic groups (as mentioned before), for the samples showing A_tot_ < content of -SO_3_H, the values of A_tot_ were considered to correspond to the amount of available active sites in the form of -SO_3_H species.

As observed in Figure 8, there was a correlation between the initial rate of glycerol consumption and the concentration of -SO_3_H sites (Figure 8A), indicating a crucial role of strongly acidic sulfonic sites in the transformation of glycerol into glycerol ethers. The density of -SO_3_H groups also had an important influence on the formation of higher substituted glycerol ethers (Figure 8B); however, both dependencies were not straightforward. In our previous studies, we found a synergistic effect of sulfonic and O-containing functional groups in carbon fibers on the formation of tert-butyl glycerol ethers [24]. Apparently, in the case of CNTs, other physicochemical or structural features of the catalysts, e.g., limited or improved availability of active sites in the case of large raw CNT aggregates or ball-milled samples, respectively (see Figure S2), could play a significant role in the process, making the active sites on the catalyst’s surface more or less accessible to the reagents [13]. 

**Figure 9:**
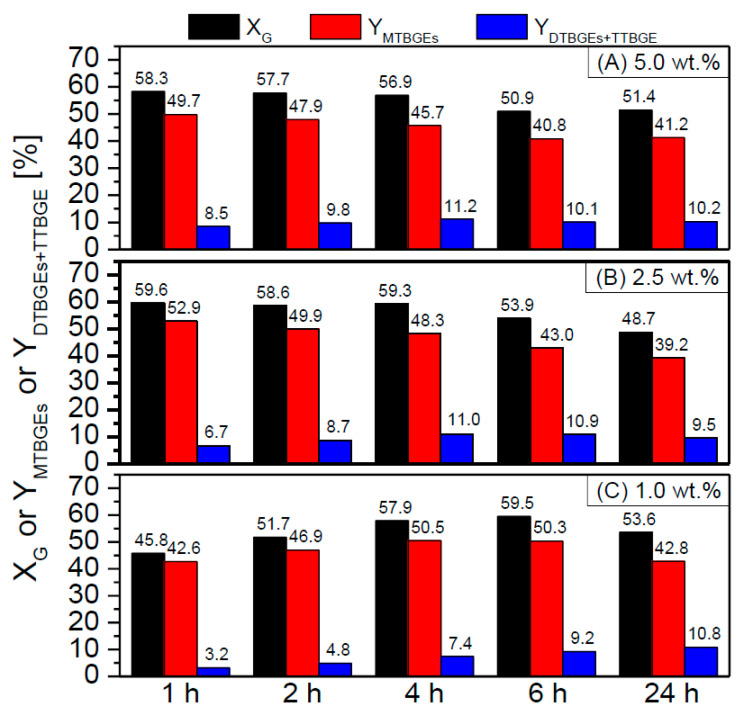
The effect of NC3100-BDS-50 catalyst loading, i.e., 5.0 wt.% (**A**), 2.5 wt.% (**B**), or 1.0 wt.% (**C**), on the glycerol etherification (temp. = 110 °C, G:TBA molar ratio = 1:4).

**Figure 10:**
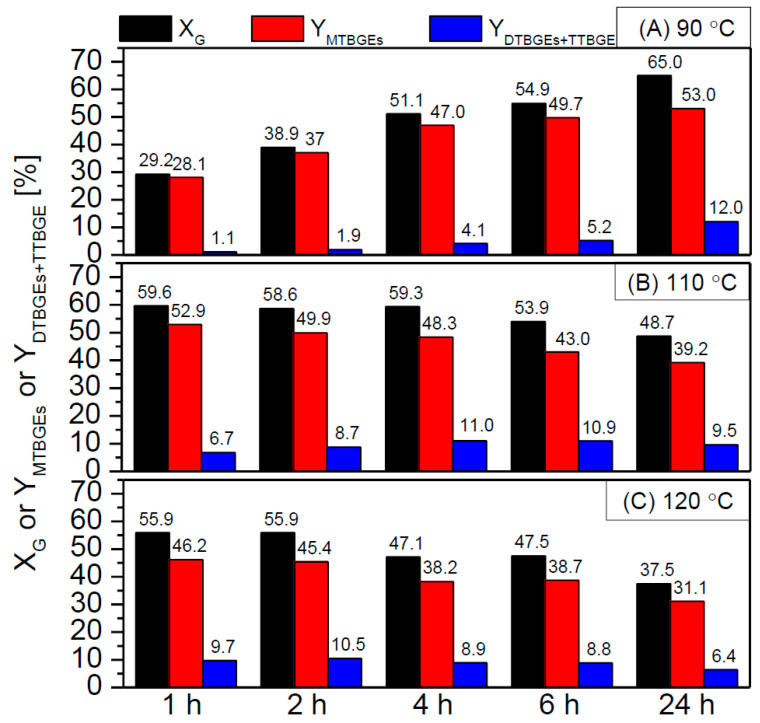
The effect of the reaction temperature, i.e., 90 °C (**A**), 110 °C (**B**), or 120 °C (**C**), on the activity of NC3100-BDS-50 in the glycerol etherification (catalyst loading = 2.5 wt.%, G:TBA molar ratio = 1:4).

For the best-performing catalyst, i.e., NC3100-BDS-50, the influence of selected reaction conditions (catalyst loading and reaction temperature) on the course of the process was studied. The obtained results are presented in Figure 9 and Figure 10.

As shown in Figure 9, there were differences in the process efficiency when using various loadings of the catalyst (i.e., 5, 2.5, or 1 wt.%); however, the changes were not very significant, especially when 5 wt.% or 2.5 wt.% of catalyst were applied. The initial glycerol conversions observed for these two cases were comparable and very high and did not change significantly with time (only a slight de-etherification effect was observed after 4 h). Moreover, promising yields of ethers were achieved within just 1 h for both catalyst concentrations. Further lowering the catalyst loading to 1 wt.% strongly affected the initial rate of the DTBGEs and TTBGE formation. After 1 h, the glycerol conversion was still quite high (~45%); however, the yield of the most desired DTBGEs and TTBGE was negligible (~3%). These results improved with extended reaction time; however, the satisfactory results were only achieved after 4 h. Thus, these data suggest that lowering the CNT catalyst loading to 2.5 wt.% could make the process more economically viable. It should also be highlighted that the results obtained in the present work are superior to the ones presented in the literature, especially taking into consideration the minimum catalysts loadings > 5 wt.% used by other laboratories to obtain satisfactory X_G_ and ether yields [57,59].

Figure 10 compares the X_G_ and ethers’ yields in the glycerol etherification performed at different temperatures, i.e., 90 °C, 110 °C, and 120 °C. 

As can be observed, the temperature used significantly impacted the catalyst performance. At a temperature of 90 °C, the catalyst worked the least effectively, giving only a 30% conversion of glycerol within 1 h and virtually producing only MTBGEs. In this case, satisfactory yields of higher-substituted products (i.e., DTBGEs+TTBGE) were achieved only after 24 h of the reaction. On the other hand, higher temperatures positively affected the process, increasing the initial X_G_ parameters and facilitating the production of DTBGEs and TTBGE. This effect was particularly visible at 120 °C when the glycerol conversion as high as ~56% was obtained and the DTBGEs+TTBGE yield reached ~10% within 1 or 2 h. Interestingly, under the above conditions, the de-etherification effect was strongly favored at longer reaction times, resulting in a drastic drop in glycerol conversion and, consequently, yields of the reaction products. The promoting effect of high temperature on the hydrolysis of glycerol ethers was also reported by Klepáčová et al. [60] and Gonçalves et al. [57]. On the other hand, performing the reaction at 120 °C allowed us to achieve excellent catalytic results in a short time, thus enabling the reduction of TBGE production costs. Moreover, the achieved results were quite impressive compared with those presented by others. For example, Carvalho et al. [61] found that the sulfonated carbons prepared from rice husk could give only ~20% glycerol conversion within 1 h, while Miranda et al. [59] achieved a glycerol conversion of only 31–35% using sulfonated activated carbons, despite applying the extended reaction time (10 h) and quite a high catalyst loading (7.5 wt.%).

The reusability of heterogeneous catalysts is one of the most important advantages of these systems. Thus, selected modified CNT samples (i.e., the best working NC3100-BDS-50 and NC7000-BM-BDS-50 catalysts) were re-used in subsequent glycerol etherification reactions. The obtained results are depicted in Figures S5 and S6 in Supplementary Materials.

As shown in Figure S5, the NC7000-BM-BDS-50 sample showed comparable catalytic performance over three consecutive reaction cycles, with only a minor drop in glycerol conversions and yields of ethers in subsequent runs. However, after the third cycle, a significant decrease in the sample activity was observed. A regeneration process of the de-activated catalyst was performed according to our previously applied method (i.e., treating the sample with a 5% HCl solution for 20 h) [33]. Interestingly, the catalytic performance of the sample was partially restored, suggesting that the deterioration of the catalytic activity was probably related to a strong interaction between the surface active sites and reagents rather than the active site leaching [33,62]. The reusability of the NC3100-BDS-50 sample is depicted in Figure S6 in the Supplementary Materials. In this case, the catalyst was stable over four reaction cycles.

## 3. Materials and Methods

### 3.1. Materials

Research- and industrial-grade multi-walled carbon nanotubes (NC3100 and NC7000, respectively) were purchased from Nanocyl SA, Sambreville, Belgium. The products varied in their purity, i.e., NC3100 represented a high purity material with above 95% C content, whereas NC7000 contained ≥ 90% of C. Sulfanilic acid (ACS, Reag. Ph. Eur.) was purchased from Merck, Germany, sodium nitrite (p.a.) and anhydrous glycerol (p.a., 99.5%) were obtained from Avantor Performance Materials, Poland, hydrochloric acid (35–38%, p.a.) was received from StanLab, Poland, whereas tert-butyl alcohol (p.a.) was purchased from Aktyn, Suchy Las, Poland. 

### 3.2. Functionalization of the Samples

NC3100 and NC7000 were functionalized with 4-benzenediazonium sulfonate (BDS) generated in situ to anchor sulfonic groups to the CNT surface and induce the sample acidity. The applied procedure was as follows: 95 mL of distilled water, 1.5 g of CNTs, and 2.1 g of sulfanilic acid were mixed for 1.5 h using an ultrasonic bath. Afterward, 0.9 g of sodium nitrite and 15 mL of concentrated hydrochloric acid (added dropwise) were put into the flask while mixing with a magnetic stirrer. The modification was performed at 20 °C or 50 °C for 20 h. The resulting samples were filtered and washed with hot distilled water, small portions of methanol, DMF, and acetone. Finally, the materials were dried at 110 °C overnight and sieved to a particle size of ≤0.4 mm. 

A selected sample (i.e., NC7000) was also subjected to mechanical pretreatment before functionalization. This was performed using a Retsch MM200 ball mill equipped with zirconium oxide milling balls for 20 min with a vibrational frequency of 25 vibrations/s. The as-prepared material (denoted as NC7000-BM) was then modified with BDS according to the procedure described above. 

The functionalized samples were labeled according to the scheme: sample type-type of modifying agent-temperature of modification. For example, NC7000-BM-BDS-50 means the ball-milled NC7000 sample was modified with 4-benzenediazonium sulfonate at 50 °C.

### 3.3. Characterization of the Samples

Quantitative C, H, N, and S elemental analysis (EA) of the samples was performed using a Flash 2000 analyzer. Ash was established as the residue after thermogravimetric (TG) analysis was performed in an air atmosphere. A potentiometric back titration method was used to determine the total acidity of the materials (i.e., the total number of Brønsted acid sites; A_tot_). For this purpose, ~0.1 g of the sample was mixed with 50 mL of a 0.01 M NaOH solution for 20 h at ambient temperature. Afterward, the carbon was filtered, and 20 mL of the filtrate was titrated with 0.05 M HCl using a CerkoLab microtitrator. High-resolution transmission electron microscopy (HRTEM) using an FEI Tecnai G2 20 X-TWIN apparatus was applied to study the morphology of the samples. Selected CNT samples were also investigated with a ZEISS EVO 40 scanning electron microscope (SEM). The textural properties of the prepared materials were analyzed by performing N_2_ adsorption/desorption measurements at −196 °C with Quantachrome Autosorb IQ equipment. The specific surface areas (S_BET_) of samples were calculated using the BET equation, while the t-plot method was applied to establish the micropore volumes (V_μ_) and external surface areas of carbons (S_ext_). The total pore volumes (V_tot_) of CNTs were measured from the amount of N_2_ adsorbed at a relative pressure close to 1. Thermogravimetric (TG) analysis was performed using a Setaram Setsys 1200 thermal analyzer. The measurements were carried out in air or nitrogen flow and the temperature range of 20 °C–1000 °C (heating rate of 10 °C/min). X-ray photoelectron spectroscopy (XPS) studies were performed using a SPECS UHV multichamber analytical system. In turn, Raman spectra were obtained by applying a Renishaw InVia Reflex confocal Raman spectrometer equipped with an argon laser as an excitation source (λ = 514 nm, P = 1 mW).

### 3.4. Catalytic Measurements

The functionalized CNTs were tested as catalysts in the etherification of glycerol (G) with tert-butyl alcohol (TBA) under autogenous pressure. In each run, 10.2 g of glycerol and 42 mL of TBA (G:TBA molar ratio of 1:4) were transferred into a stainless-steel autoclave and stirred for complete homogenization. Afterward, catalyst (5, 2.5, or 1 wt.% based on the glycerol weight) was added to the mixture, and the reactor was sealed and flushed with Ar. The catalytic testing was performed at 90, 110, or 120 °C for 24 h. To monitor the progress of the reaction, aliquots of the reaction mixture were periodically taken out (after 1, 2, 4, 6, and 24 h), diluted with isopropanol, and analyzed using a SRI 8610C gas chromatograph equipped with a RESTEK MXT^®^—WAX capillary column (30 m × 0.25 mm × 0.25 μm), a split injector, and FID detector. The catalytic activity of the samples was expressed as the conversion of glycerol (X_G_) and yields of individual reaction products (i.e., mono-, di-, and tri-tertbutyl glycerol ethers; MTBGEs, DTBGEs, and TTBGE, respectively). Reusability tests were also performed on selected samples. For this purpose, the spent catalyst was recovered from the reaction mixture by filtration, washed with hot distilled water, then acetone, and dried. After sieving, the catalyst was re-used under standard reaction conditions. The catalyst regeneration was performed by mixing the sample with a 5% HCl solution for 20 h, then washing it with hot distilled water and drying at 110 °C overnight. The reproducibility of all catalytic tests was very high, and the standard deviations calculated for glycerol conversions and ether yields were lower than 1.5%.

## 4. Conclusions

A facile method of obtaining highly active solid acid CNTs was proposed using 4-benzenediazonium sulfonate generated in situ. This strategy introduced significant amounts of -SO_3_H species onto the surface of CNTs, leading to a significant increase in the total acidity (A_tot_) of these catalysts. It was demonstrated for the first time that the functionalization of chemically stable CNTs can be facilitated by a simple pretreatment of the sample using ball-milling, which fragmented the aglomerates and resulted in a generation of new “active” edges susceptible to functionalization. Moreover, the functionalization of CNTs with BDS was favored at higher temperatures, resulting in the introduction of higher quantities of sulfur into the materials’ structure. The proposed functionalization method selectively introduced sulfur in the form of sulfonic groups, known to be very active Brønsted acid sites.

The resulting sulfur-doped CNTs were tested in glycerol valorization to substituted ethers. All the prepared samples showed high activity in etherification due to their highly functionalized surface chemistry. The catalytic performances of the most active catalysts (i.e., NC3100-BDS-50 and NC7000-BM-BDS-50) exceed those of the commercial Amberlyst-15 catalyst. Under optimized reaction conditions, a remarkable ~56% glycerol conversion and ~10% yield of DTBGEs+TTBGE was obtained within just 1 h of the reaction. The catalyst acidity induced by the -SO_3_H groups was found to be a key factor for the efficient transformation of glycerol to glycerol ethers. Furthermore, the developed BDS-modified CNTs solid acid catalysts showed excellent recyclability, opening up new possibilities for the application of these materials in other acid-catalyzed industrial processes in the future. 

## Figures and Tables

**Table 1:** The results of the total acidities determinations (A_tot_) and content of -SO_3_H groups obtained for the tested materials.

Parameter	NC3100	NC7000	NC7000-BM	NC3100-BDS-50	NC7000-BDS-20	NC7000-BDS-50	NC7000-BM-BDS-50
A_tot_ [mmol H^+^/g]	0.02	0.03	0.05	1.01	0.38	0.66	1.16
content of -SO_3_H groups * [mmol/g]	0.00	0.00	0.00	1.05	0.37	0.83	0.94

* Calculated based on EA results.

**Table 2:** The contents of carbon, sulfur, and oxygen determined for the selected samples.

Sample	C [wt.%]	O [wt.%]	S [wt.%]
NC7000-BDS-20	89.86	6.20	3.90
NC7000-BDS-50	87.02	8.40	4.60
NC7000-BM-BDS-50	81.88	11.30	6.80

**Table 3:** The relative contributions of various S- and O-species calculated from XPS S 2p and O 1s profiles.

	S 2p	O 1s		
**Sample**	**S** in SO_3_H [at.%]	**O** in C=O, S=O [at.%]	**O** in C-O, S-O [at.%]	**O** in COOH [at.%]
NC7000-BDS-20	100.00 (3.90 wt.%)	69.70 (4.32 wt.%)	22.20 (1.38 wt.%)	4.30 (0.26 wt.%)
NC7000-BDS-50	100.00 (4.60 wt.%)	68.90 (5.78 wt.%)	24.60 (2.06 wt.%)	4.30 (0.36 wt.%)
NC7000- BM-BDS-50	100.00 (6.80 wt.%)	69.10 (7.80 wt.%)	23.70 (2.67 wt.%)	4.10 (0.46 wt.%)

## Data Availability

The data presented in this study are available on request from the corresponding authors.

## Supplementary Materials

The following supporting information can be downloaded at: https://www.mdpi.com/article/10.3390/molecules29071623/s1; Figure S1. General scheme of CNTs modification with 4-benzenediazonium sulfonate, indicating some possible surface structures formed [36,37]; Figure S2: SEM images of (A) raw and (B) ball-milled NC7000 sample at different magnifications; Figure S3: The DTG patterns obtained for the raw NC7000 and NC3100 samples under an air gas flow; Figure S4: Reaction scheme of glycerol etherification with tert-butyl alcohol under acidic conditions; Figure S5: The results of the reusability tests performed for NC7000-BM-BDS-50 (temp. = 110 °C, catalyst loading = 5 wt.%, G:TBA molar ratio = 1:4); Figure S6: The results of the reusability tests performed the NC3100-BDS-50 (temp. = 110 °C, catalyst loading = 5 wt.%, G:TBA molar ratio = 1:4).
