# Supplementary material for: Promoting Effect of Ball Milling on the Functionalization and Catalytic Performance of Carbon Nanotubes in Glycerol Etherification
Source: Molecules. 2024 Apr 4;29(7):1623. doi: 10.3390/molecules29071623 (PMC11013610; doi:10.3390/molecules29071623)
Supplement: Supplementary file 1 [file molecules-29-01623-s001.zip › molecules-2919204-supplementary.pdf]

Supplementary Materials

Promoting Effect of Ball Milling on the Functionalization and Catalytic Performance of Carbon Nanotubes in Glycerol Etherification

Karolina Ptaszyńska [1,*], Anna Malaika [1], Katarzyna Morawa Eblagon [2,3], José Luís Figueiredo [2,3] and Mieczysław Kozłowski [1,*]

1 Faculty of Chemistry, Adam Mickiewicz University in Poznań, Uniwersytetu Poznańskiego 8, 61-614 Poznań, Poland; amalaika@amu.edu.pl (A.M)
2 LSRE-LCM—Laboratory of Separation and Reaction Engineering—Laboratory of Catalysis and Materials, Faculty of Engineering, University of Porto, Rua Dr. Roberto Frias, 4200-465 Porto, Portugal; keblagon@fe.up.pt (K.M.E.); jlfig@fe.up.pt (J.L.F.)
3 ALiCE—Associate Laboratory in Chemical Engineering, Faculty of Engineering, University of Porto, Rua Dr. Roberto Frias, 4200-465 Porto, Portugal
* Correspondence: karolina.ptaszynska@amu.edu.pl (K.P.); mkozlow@amu.edu.pl (M.K.).

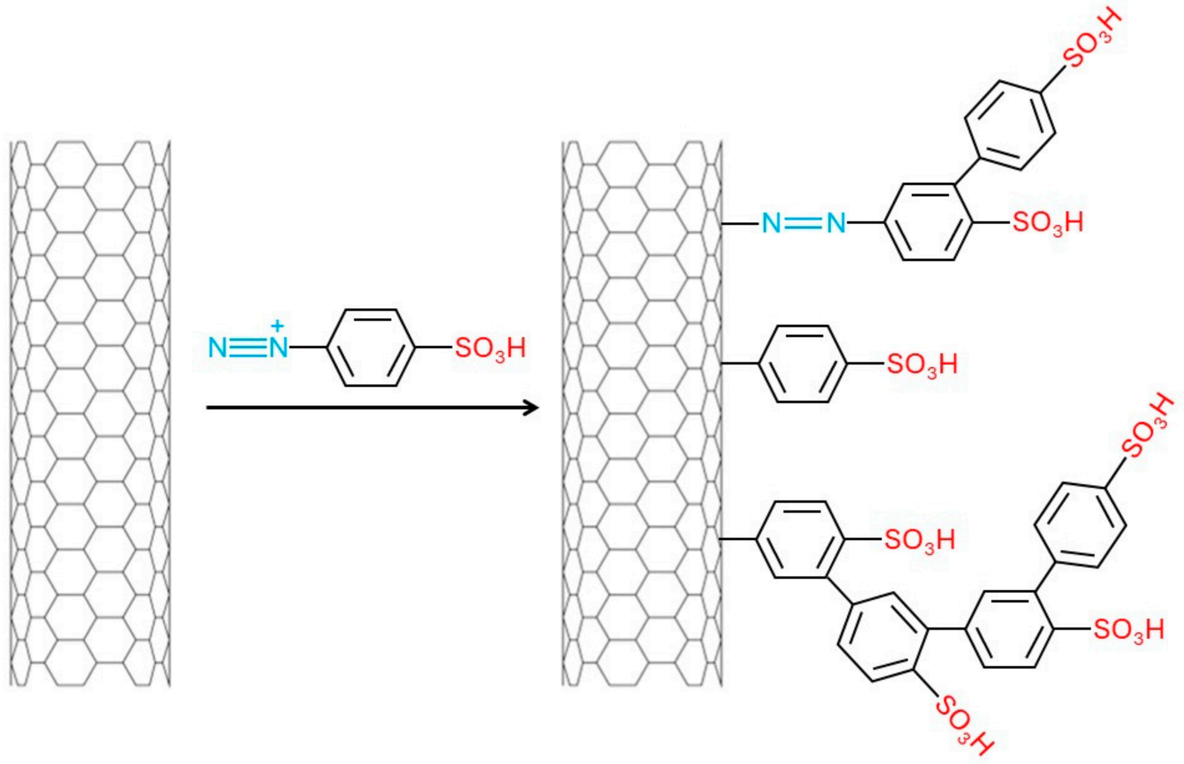

Figure S1. General scheme of CNTs modification with 4-benzenediazonium sulfonate, indicating some possible surface structures formed (based on [36,37]).

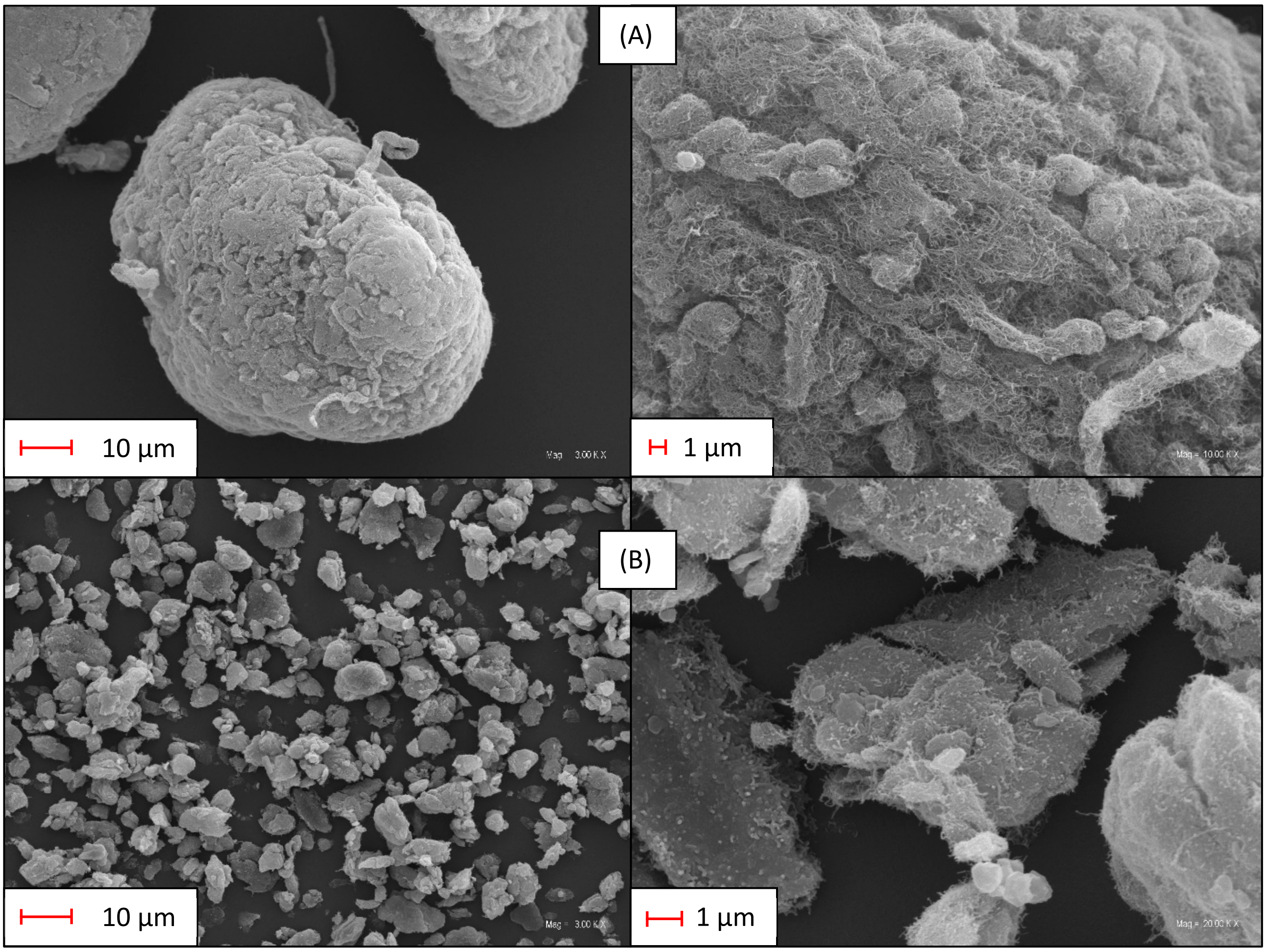

Figure S2. SEM images of (**A**) as-received and (**B**) ball-milled NC7000 samples at different magnifications.

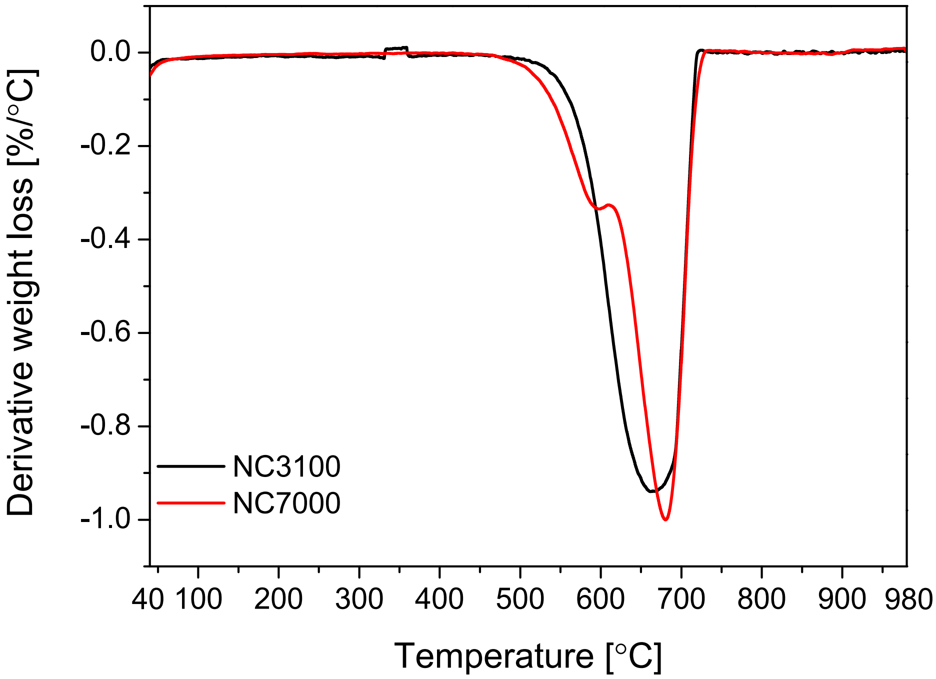

Figure S3. The DTG patterns obtained for the raw NC7000 and NC3100 samples under an air gas flow.

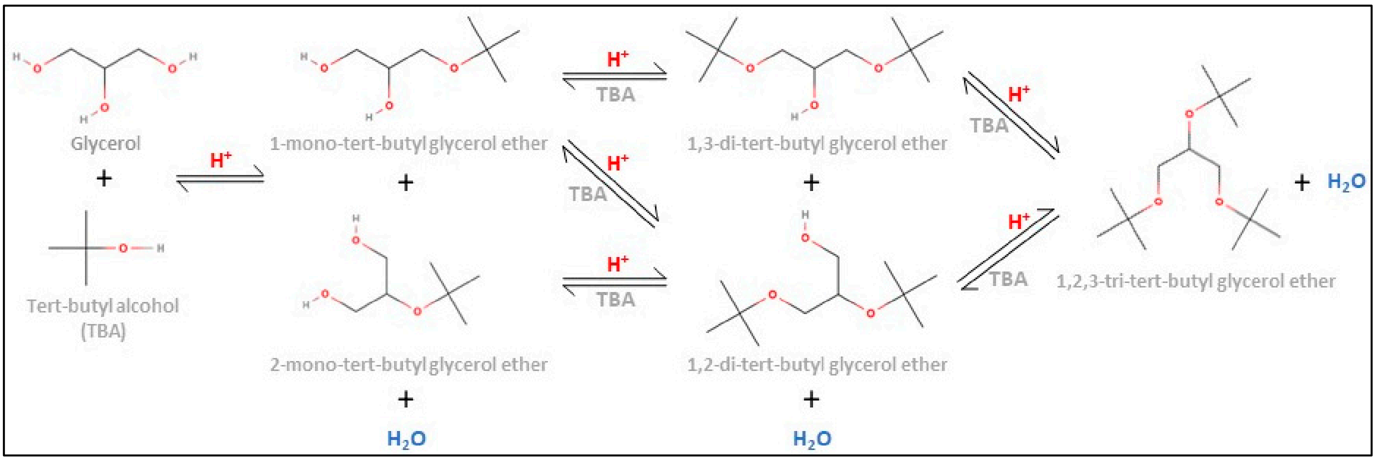

Figure S4. Reaction scheme of glycerol etherification with tert-butyl alcohol under acidic conditions.

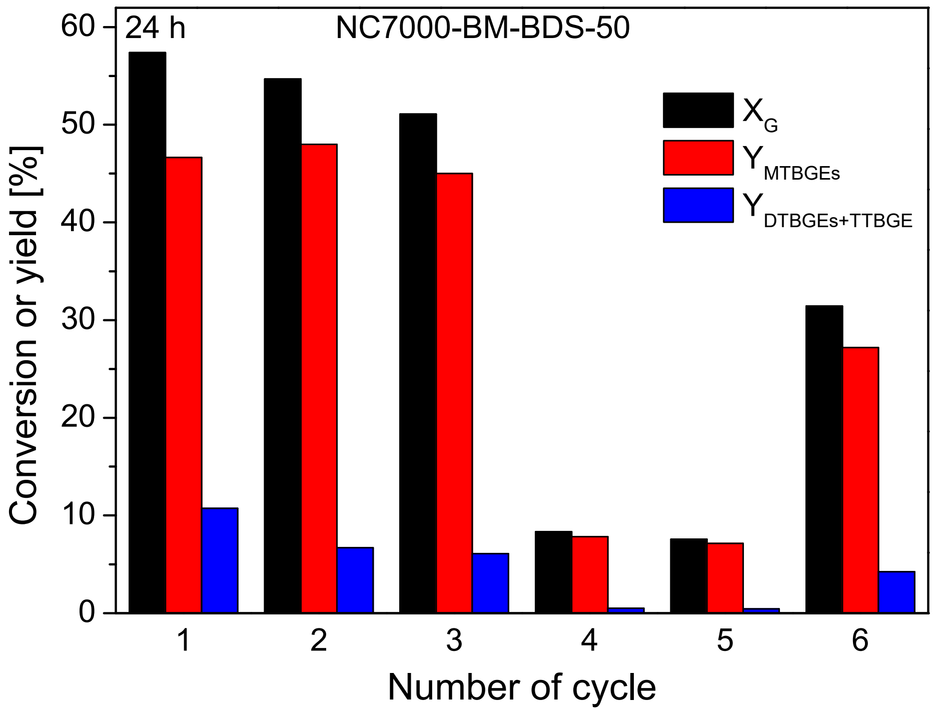

Figure S5. The results of the reusability tests performed for the NC7000-BM-BDS-50 (temp. = 110 °C, catalyst loading = 5 wt.%, G:TBA molar ratio = 1:4).

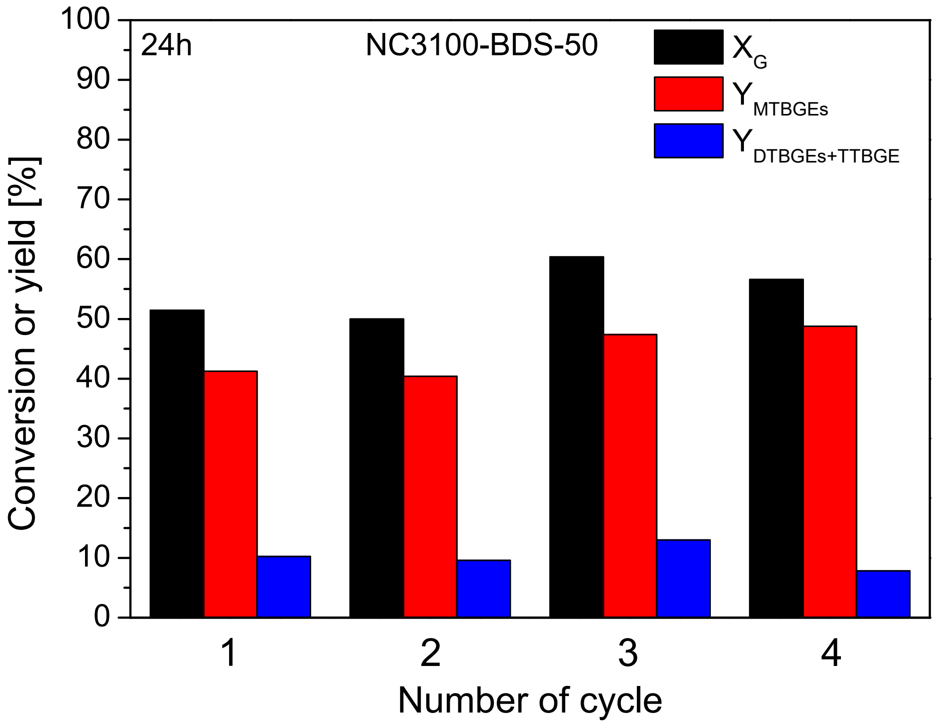

Figure S6. The results of the reusability tests performed for the NC3100-BDS-50 (temp. = 110 °C, catalyst loading = 5 wt.%, G:TBA molar ratio = 1:4).
